# Stigmatizing Beliefs About Alzheimer’s Disease in Diverse Ethnic Groups of Asian Americans

**DOI:** 10.1093/geroni/igaa057.888

**Published:** 2020-12-16

**Authors:** Jiaming Liang, Yuri Jang

**Affiliations:** University of Southern California, Los Angeles, California, United States

## Abstract

Cumulative studies have investigated Alzheimer’s disease (AD)-related issues among Asian Americans, but few have considered ethnic diversities within the Asian group. Using an ethnic-diverse Asian American sample, the present study explored the prevalence, ethnic variations, and predictors of stigmatizing beliefs about AD: (1) AD is a normal process of aging, (2) it is embarrassing to have a family member with AD, and (3) social interactions with an AD patient should be avoided. Inspired by the sociocultural health beliefs model, a focus was given on the role of immigration and culture-related variables. Using data from the 2015 Asian American Quality of Life survey (N = 2609, age range = 18-98) that includes Chinese, Asian Indian, Korean, Vietnamese, Filipino, and other Asians, logistic regression was conducted to examine how each of the three stigmatizing beliefs would be predicted by (1) demographic variables and (2) immigration and culture-related variables. Results indicate that the prevalence of the stigmatizing beliefs about AD varied across ethnicities. More than 63% of Vietnamese associated AD with a normal process of aging, and about 10% of Chinese reported that they would feel embarrassed if their family member had AD. Logistic regression models demonstrated that advanced age, male gender, low education, and limited English proficiency increased the odds of reporting one or multiple stigmatizing beliefs about AD. The findings suggest a varying degree of AD-related misconceptions and stigmatization and call attention to the need for culturally sensitive community education on AD in Asian communities.

